# A Study on the Utilization of Coal Fly Ash Derived Grog in Clay Ceramics

**DOI:** 10.3390/ma13225218

**Published:** 2020-11-18

**Authors:** Thye Foo Choo, Mohamad Amran Mohd Salleh, Kuan Ying Kok, Khamirul Amin Matori, Suraya Abdul Rashid

**Affiliations:** 1 Institute of Advanced Technology, University Putra Malaysia, UPM Serdang, Selangor 43400, Malaysia; ctfoo@nuclearmalaysia.gov.my (T.F.C.); khamirul@upm.edu.my (K.A.M.); suraya_ar@upm.edu.my (S.A.R.); 2 Malaysian Nuclear Agency (Nuclear Malaysia) Bangi, Kajang, Selangor 43000, Malaysia; kyk1000@nuclearmalaysia.gov.my; 3 Department of Chemical and Environmental Engineering, University Putra Malaysia, UPM Serdang, Selangor 43400, Malaysia; 4 Department of Physics, Faculty of Science, University Putra Malaysia, UPM Serdang, Selangor 43400, Malaysia

**Keywords:** grog, coal fly ash, clay ceramics, waste utilization

## Abstract

Grog is an additive material that plays important roles in ceramic making. It improves the fabrication process of green bodies as well as the physical properties of fired bodies. Few low-cost materials and wastes have found their application as grog in recent years, thus encouraging the replacement of commercial grogs with cost-saving materials. Coal fly ash, a combustion waste produced by coal-fired power plant, has the potential to be converted into grog owing to its small particle sizes and high content of silica and alumina. In this study, grog was derived from coal fly ash and mixed with kaolin clay to produce ceramics. Effects of the grog addition on the resultant ceramics were investigated. It was found that, to a certain extent, the grog addition reduced the firing shrinkage and increased the total porosity of the ceramics. The dimensional stability of the ceramics at a firing temperature of 1200 °C was also not noticeably affected by the grog. However, the grog addition in general had negative effects on the biaxial flexural strength and refractoriness of the ceramics.

## 1. Introduction

Grog traditionally refers to a small granular material composed of aggregate made from crushed brick or other fired ceramic. Grog, a non-plastic material, is usually added to ceramic green bodies to improve the fabrication process as well as the physical properties of the fired bodies [1]. In the fabrication process, grog is used to increase pore space in the green body to enable quicker venting of water vapor during drying and gases of decomposition during firing [1,2]. Grog is also used to reduce shrinkages [3]. Given that grog is normally pre-fired, its body in general does not undergo a drying shrinkage or firing shrinkage. Grog can also be used to improve fired abrasion resistance, reduce thermal expansion, reduce density and give distinct visual characteristics to the fired bodies [4,5].

Currently, grogs are produced by crushing certain natural minerals or by calcining mineral and grinding it into small aggregates. Although commercial grogs are widely available in the market, they are relatively expensive. This has led to more efforts being directed toward the development of new economical alternative materials. A variety of materials with potential use as grog have been investigated, for example, fired brick waste [1], fired kaolin clay [2], mullite [4,5], cordierite-mullite [6], bauxite [7] and blast furnace slag [8].

Coal fly ash (CFA) is a combustion by-product of coal in coal-fired thermal power plant. It has high content of silica (20–80 wt %) and alumina (1–55 wt %) [9] with traces of transition metal oxides [10]. CFA mainly consists of an amorphous aluminosilicate glassy phase and crystalline phases, namely quartz (SiO_2_) and mullite (3Al_2_O_3_·2SiO_2_). Globally, it is estimated that 750 million tons of CFA are produced each year. However, less than 50% of CFA is being utilized [11]. Currently, most of the CFA is being utilized as a substitute for fine aggregates in the construction industry, geopolymers, soil amelioration, ceramics, catalysis, adsorbent and filler [10,12]. CFA seems to be a potential candidate for grog owing to its high content of useful silica and alumina. Other than this, CFA in its fine particle form does not require additional heavy crushing and grinding processes. Waste utilization of CFA as grog in ceramic making can also bring significant benefits for the environment and ceramic industry.

In this context, the general objective of this study is to investigate the feasibility of using CFA as grog in the preparation of kaolin clay ceramic. In this study, grog was first derived from the CFA by high-temperature firing and subsequently mixed with kaolin clay. The mixtures were then uniaxially pressed and fired to produce kaolin clay ceramics. The obtained ceramics were characterized in detail and the properties of the ceramics were correlated with the addition of the CFA derived grog (CFAG).

## 2. Materials and Methods

### 2.1. Preparation of Kaolin Clay Ceramics

**Figure 1:**
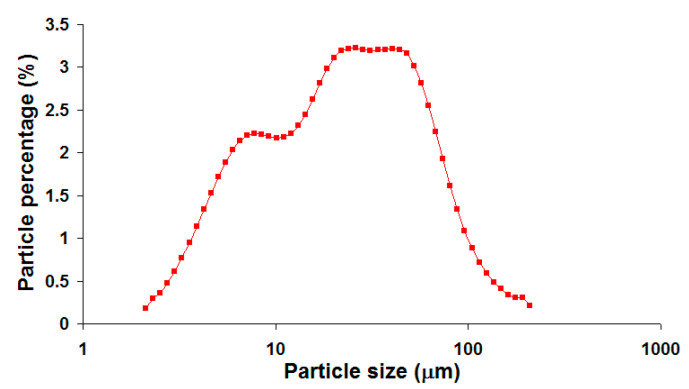
Particle size distributions (logarithmic scale on *x*-axis) of the CFA.

The raw material CFA was obtained from Sultan Salahuddin Abdul Aziz coal-fired power plant located in Kapar, Malaysia (coordinates: 3.117471,101.321842). The power plant consists of pulverized coal boilers manufactured by Ishikawajima-Harima Heavy (IHI), (Tokyo) Japan, and the coal feedstock was mainly imported from Australia and Indonesia. The kaolin clay (AKIMA-35) was obtained from Associated Kaolin Industries Co., (Petaling Jaya) Malaysia. Both crystalline and amorphous phases were detected in the CFA. The major crystalline phases identified were quartz and mullite [13]. The crystalline phases of kaolin clay were kaolinite (Al_2_O_3_·2SiO_2_·2H_2_O), quartz and a small amount of muscovite (mica) phase [14]. Table 1 lists the chemical compositions of CFA and kaolin clay, which were reported in our previous works [13,15]. The particle size distribution of the CFA as shown in Figure 1 was reported in previous work [16]. The CFA has a very broad bimodal size distribution ranging from approximately 2 µm to slightly over 200 µm and an average particle size (d50) of 21.83 µm.

Firstly, the as-received CFA was fired at 1000 °C for 2 h. The purpose of the firing was to burn out and dissociate unwanted chemical substances such as carbon in the CFA. These substances could emit gases and cause bloating in ceramic at high temperature. The pre-firing was also aimed at thermally stabilizing the CFA and reducing its firing shrinkage. After the firing, lightly agglomerated CFAG was loosened with a hand mortar. Kaolin clay was then thoroughly mixed together with 10 wt %, 20 wt %, 30 wt %, 40 wt %, 50 wt %, 60 wt %, 70 wt %, 80 wt % and 90 wt % of CFAG. The obtained mixtures were uniaxially pressed into pellets of 4 cm in diameter under 141.5 MPa compression pressure using a laboratory hydraulic press and fired at 1200 °C for 4 h to produce kaolin clay ceramics. The firing processes were conducted at a rate of 10 °C/min in an electrical box furnace and cooled to room temperature by natural convection inside the furnace. For comparison, 100 wt % of kaolin clay and 100 wt % of CFAG were also used to prepare ceramics by the same processing method. For identification purposes, the ceramic samples were named as K, KC10, KC20, KC30, KC40, KC50, KC60, KC70, KC80, KC90 and C, which corresponded to 0 wt %, 10 wt %, 20 wt %, 30 wt %, 40 wt %, 50 wt %, 60 wt %, 70 wt %, 80 wt %, 90 wt %, 100 wt % of CFAG contained in the samples. The batch composition of the mixtures is given in Table 2.

### 2.2. Characterization

Chemical compositions of the raw materials were determined by Shimadzu EDX-7000 energy dispersive X-ray fluorescence (EDXRF) (Shimadzu Corporation, Kyoto, Japan). Zeiss GeminiSEM 500 field emission scanning electron microscope (FESEM) (Zeiss, Oberkochen, Germany) was used for the study of the morphology of the fired ceramics. X-ray diffraction (XRD) patterns of the samples were recorded by using PANalytical X’Pert PRO (PANalytical Inc, Almelo, Netherlands) using monochromated CuKα radiation (λ = 1.54184 Å) and phase composition was analyzed by Rietveld refinement method [17] using X’pert HighScore Plus version 2.2b (PANalytical Inc, Almelo, Netherlands). Particle size distributions of the powders were measured by Microtrac X100 laser particle size analyzer (Microtrac, York, PA, USA). Linear thermal expansion was measured by Netzsch DIL 402C Dilatometer (Netzsch, Selb, Germany) from room temperature to 1000 °C at a heating rate of 5 °C/min. Biaxial flexural strength tests were performed with an Instron Universal Testing Machine (model 4310) (Instron, Norwood, USA). Whiteness was measured by Konica Minolta CM-2500d Spectrophotometer (Konica Minolta, Tokyo, Japan) that was calibrated using a White Calibration Plate (CM-A146). Bulk density was calculated by dividing the measured mass by the measured volume of the ceramic sample. In order to measure particle density (true density) of a sample, the sample was first finely milled to open up all its closed porosity. A known weight of this fine powder was then used to displace liquid within a pycnometer to determine its true volume and thereby its particle density [18]. Total porosity can then be calculated from bulk density and particle density using Equation (1) [19]. Open porosity (apparent porosity) was determined by the Archimedes method. The total porosity is the sum of open porosity and closed porosity, and this enables the closed porosity to be calculated.

Total porosity = (1 − (bulk density/particle density)) × 100(1)

## 3. Results and Discussion

**Figure 2:**
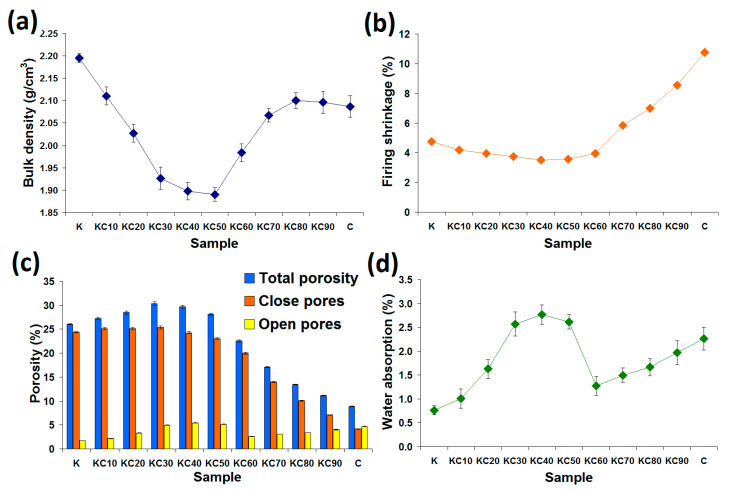
Physical properties of the fired ceramics as a function of CFAG content: (**a**) bulk density, (**b**) firing shrinkage, (**c**) porosities and (**d**) water absorption.

Figure 2 shows the plots of bulk density, firing shrinkage and porosity of the fired ceramics as a function of CFAG content. Sample K (kaolin clay with no CFAG addition) has the highest bulk density of 2.20 g/cm^3^ (Figure 2a). The bulk density gradually decreased with the addition of CFAG until the lowest value was reached at 1.89 g/cm^3^ for the sample KC50. This decrease in bulk density may be due to the porosity of CFAG aggregates and the pore space created by the contacts made between CFAG irregular-shaped aggregates and the kaolin clay particles. However, in contrast, the opposite trend was observed with the further addition of CFAG beyond 50 wt %. This was because CFAG aggregates have a higher tendency to fuse among themselves compared to the kaolin clay particles, as evidenced by the highest firing shrinkage (Figure 2b) and the lowest total porosity (Figure 2c) of sample C (100 wt % CFAG).

Firing shrinkage of the fired ceramics is shown in Figure 2b. The result shows that CFAG addition slightly decreases the firing shrinkage when the amount is 60 wt % or below. The highest reduction was from 4.75% to 3.5% with an addition of 40 wt % CFAG. CFAG which was pre-fired at 1000 °C does not undergo further firing shrinkage at temperatures below 1000 °C. This mitigates the firing shrinkage that is caused by phase transformations of kaolin clay. Yamuna et al. [20] reported that firing shrinkages of kaolin clay occur at temperatures between 450 °C and 600 °C, which was associated with the transformation of kaolinite (Al_2_O_3_·2SiO_2_·2H_2_O) to metakaolinite (Al_2_O_3_·2SiO_2_), and at 900 °C to 1000 °C due to the metakaolinite decomposition to form spinel (2Al_2_O_3_·3SiO_2_) or mullite (3Al_2_O_3_·2SiO_2_). A drastic increase in firing shrinkage was evidenced in samples with 70 wt % or higher content of CFAG (Figure 2b). The high firing shrinkage was attributed to the depletion of the amorphous phase as the result of devitrification of silica and crystallization of mullite. The highest firing shrinkage was recorded in sample C (10.75%).

Figure 2c shows the percentage of closed pores and open pores, as well as the total porosity of the fired ceramics. The total porosity of the fired ceramics was initially increased with increasing CFAG content. The highest value of 30.34% was achieved by an addition of 30 wt % CFAG (KC30), which represents a 4.26% increase over sample K. However, the total porosity subsequently dropped to 8.86% as CFAG content reached 100 wt % (a 17.22% drop compared to sample K). Other than this, it was found that the pores in the fired ceramics consisted mainly of closed pores except for sample C. The results also reveal that CFAG addition has minimal impact on the open pores compared to the closed pores.

Water absorption is an important parameter that characterizes a ceramic, especially in tile making. Generally, ceramics that have low water absorption, such as porcelain, are more desirable because of the higher durability and strength, as water absorbed into the ceramic body may cause failure by cyclic salt attack and freeze–thaw cycle [21]. The water absorption percentage also reflects the bulk density and, hence, the strength of the ceramic body. Figure 2d shows that sample K has the lowest water absorption of 0.76%. As observed in the same figure, the percentage of water absorption was dependent on the open pores of the samples. According to ISO 13006:1998 [22], ceramic tiles are divided into four categories based on their water absorption capacities: high water absorption of more than 6.0% (B2b non-vitreous), medium water absorption of more than 3.0% but less than 6.0% (B2a semi-vitreous), low water absorption of more than 0.5% but less than 3.0% (B1b fully Vitrified) and very low water absorption of less than 0.5% (B1a impervious). Figure 2d shows that all the fired ceramics have low water absorption which falls within the range of 0.5 to 3.0%; thus, they are categorized as “B1b fully vitrified” and can be considered as good candidates for tile making.

**Figure 3:**
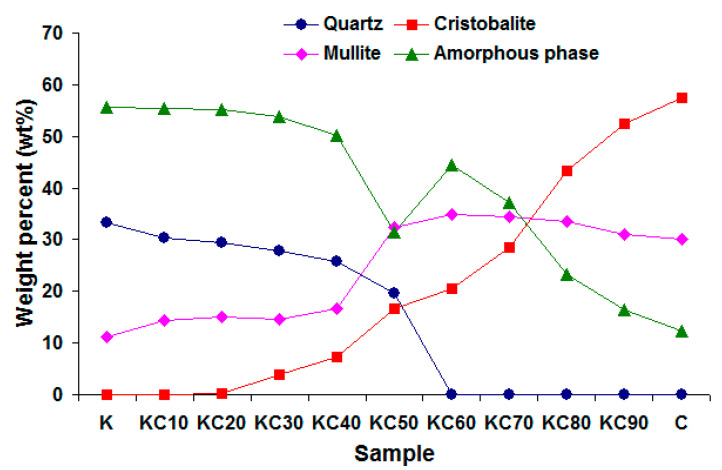
Mineralogical compositions of the ceramics fired at 1200 °C.

The mineralogical compositions of the ceramics that were fired at 1200 °C are given in Figure 3. The results were computed using diffraction data analysis software X’pert HighScore Plus version 2.2b (PANalytical Inc, Almelo, Netherlands). The software was used to profile fit the diffraction data, and the goodness of fit (GOF) values of the fitting ranged from 3.21 to 3.62. The result shows that CFAG addition has significant impacts on the mineralogy of the fired ceramics. Firstly, the mullite crystalline phase is noticeably higher in the fired ceramics with a CFAG content of 50 wt % and above. Besides the fact that CFAG contained higher amount of mullite than the kaolin clay after firing at 1200 °C, the minor oxides (Fe_2_O_3_ and TiO_2_) found in CFAG are believed to have played an important role in the growth of mullite crystals. Fe_2_O_3_ and TiO_2_ have been reported to be able to lower the activation energy of the reaction and enhance the reaction rate of Al_2_O_3_-SiO_2_ to form mullite [23]. Secondly, the cristobalite (SiO_2_) crystalline phase in the samples increased with increasing CFAG addition. Cristobalite is a silica polymorph that can be transformed from quartz. Although kaolin clay has a substantial amount of quartz, the quartz was not transformed to cristobalite during firing at 1200 °C. The quartz from kaolin clay only changes to cristobalite upon firing at temperatures between 1200 °C and 1400 °C [24]. On the other hand, quartz from CFA has a higher ability to transform to cristobalite at temperatures below 1200 °C. This is attributed to the alkali metal oxides found in CFA that can accelerate the devitrification rate of silica into cristobalite [25]. This explained the high content of quartz in kaolin-rich samples and high content of cristobalite in CFAG-rich samples. Thirdly, the result shows a general decrease in the amorphous phase from sample K to C. The decrease was due to the lower amount of amorphous phase present in CFAG when fired at 1200 °C. In the same figure, a sharp decrease in amorphous phase can be seen in sample KC50. This was attributed to the large increments of the crystalline mullite (from 16.6 wt % in KC40 to 32.3 wt % in KC50) and cristobalite (from 7.4 wt % in KC40 to 16.6 wt % in KC50) phases as well as the large amount of quartz (19.5 wt %) that remained unvitrified in the sample. The full vitrification of quartz (0 wt % quartz) seen in sample KC60 subsequently increased the amorphous phase.

**Figure 4:**
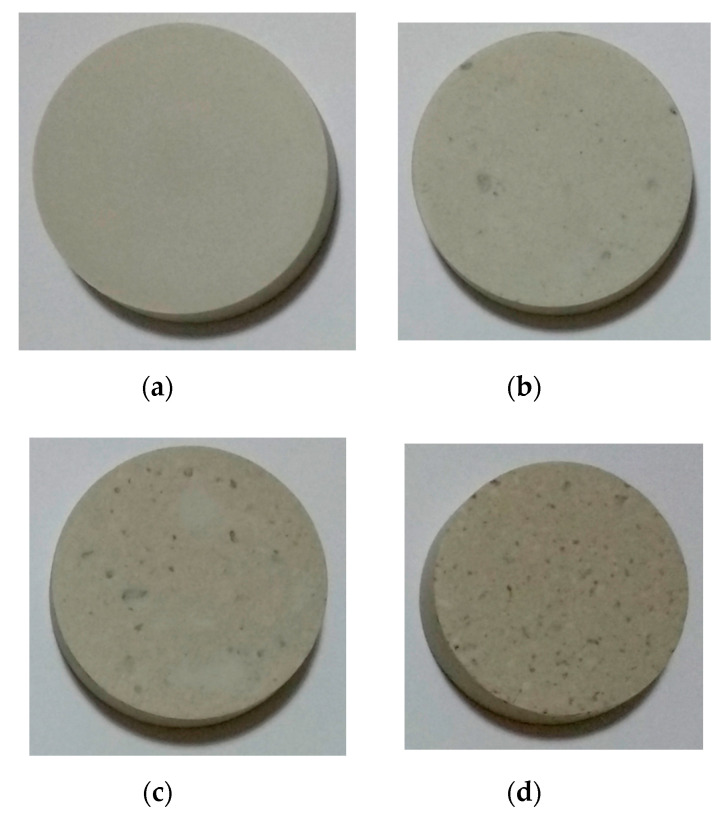

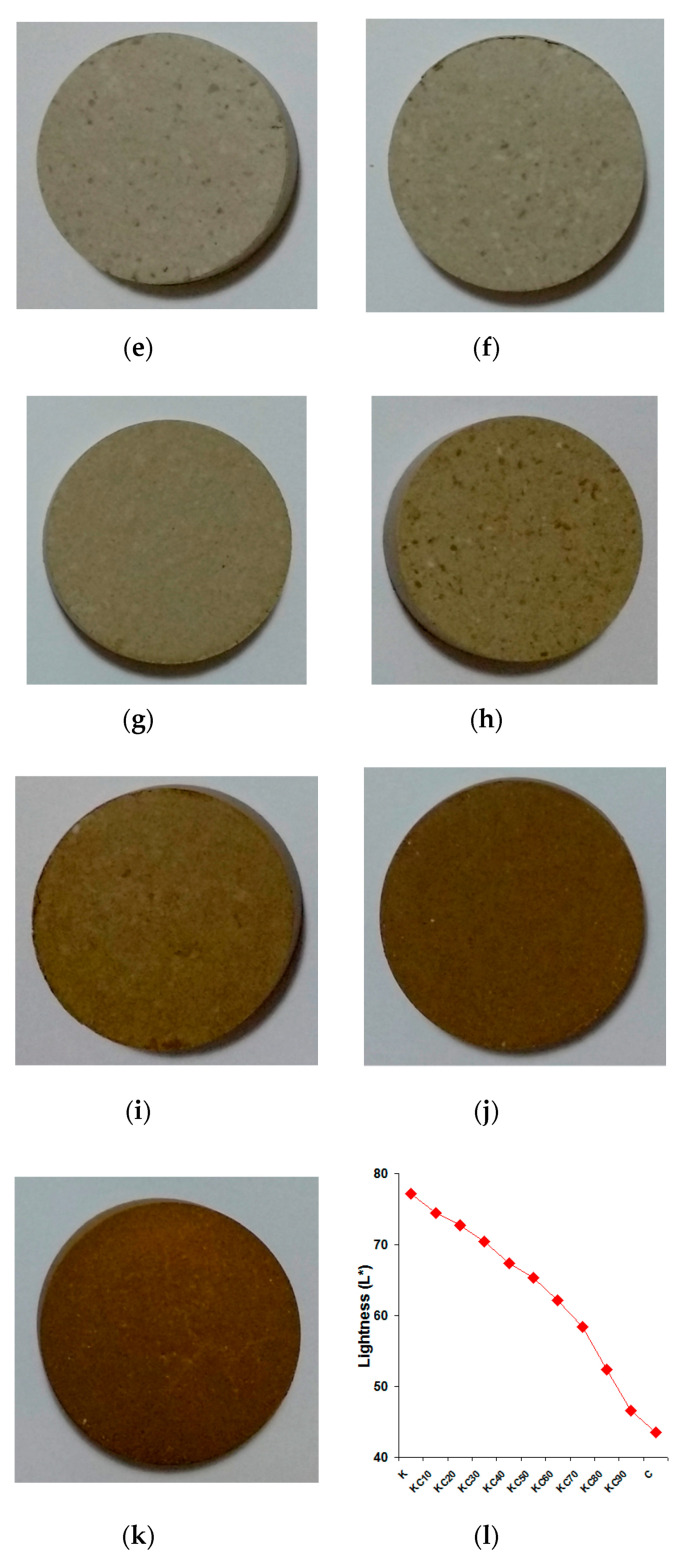
Macromorphology of (**a**) K; (**b**) KC10; (**c**) KC20; (**d**) KC30; (**e**) KC40; (**f**) KC50; (**g**) KC60; (**h**) KC70; (**i**) KC80; (**j**) KC90; (**k**) C and (**l**) lightness (L*) of the fired ceramics.

The macromorphology of the fired ceramics is shown in Figure 4. All the obtained ceramics with various amounts of CFAG have good dimensional stability at the firing temperature of 1200 °C. No cracks and no signs of melting such as bloating and deformation were noticed in the samples. However, different degrees of shrinkage were observed in samples with high CFAG content, which is measured and represented in Figure 2b.

The ceramics were also subjected to whiteness measurement indicated by lightness (L*) [26]. The result is graphically represented in Figure 4l. The figure shows that the whiteness of the samples decreased with increasing CFAG addition. This was attributed to the color-forming minor oxides present in CFA such as K_2_O, Fe_2_O_3_ and TiO_2_ [27]. Sample K has the highest L* value of 77.2 and the value reduced to 43.5 as CFAG content reached 100 wt %. It should be noted that sample K showed the highest value due to the presence of lower amounts of the mentioned oxides compared to the others. From a different perspective, when comparing the surface appearance of sample K and the others in Figure 4, it is obvious that the surface of sample K is notably plain. Contrarily, the other samples have more natural and rustic visual characteristics.

**Figure 5:**
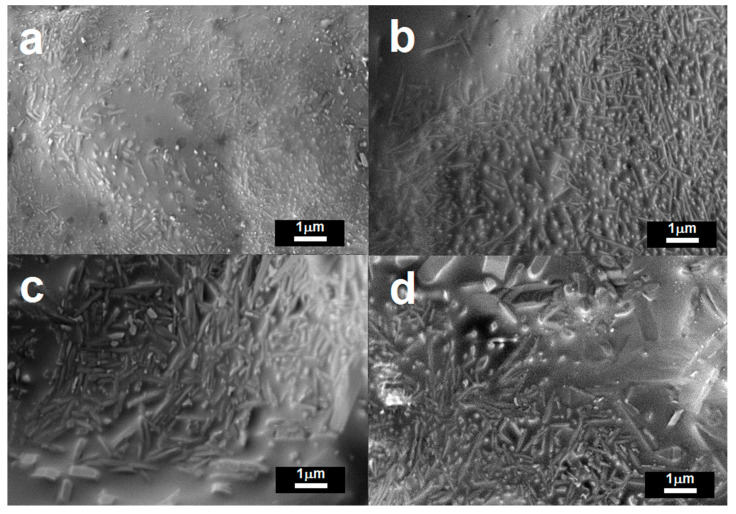
FESEM micrographs of the surface of the fired ceramic sample, (**a**) K; (**b**) KC50; (**c**) KC60 and (**d**) C.

Figure 5 shows the FESEM micrographs of the samples. Figure 5a shows that sample K consists of a number of crystals embedded in an amorphous glass matrix. The crystals are a combination of easily distinguishable acicular mullite crystals as well as quartz crystals of different sizes and shapes. The mullite crystals were nucleated and grown from the thermal decomposition of kaolinite [20]. The microstructure of KC50 is shown in Figure 5b, and considerable growth of high aspect ratio mullite crystals is observed. Overall, the amount of mullite crystals is apparently higher in those fired ceramics with CFAG addition; this observation agrees well with the mineralogical compositions of the samples (Figure 3). Relatively larger crystals with low aspect ratio can be observed in samples with high CFAG content, for example, KC60 and C as shown in Figure 5c,d. These crystals are suggested to be cristobalite (Figure 3). Therefore, it can be concluded that CFAG addition promotes the growth of mullite and cristobalite crystals.

**Figure 6:**
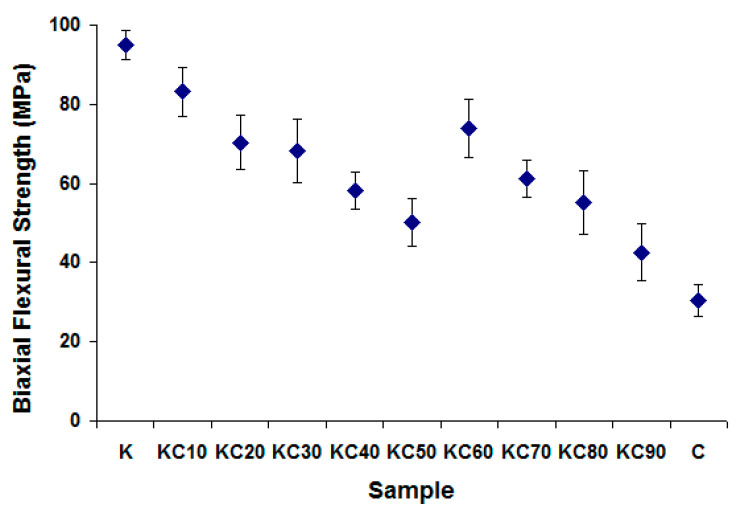
The biaxial flexural strength of the fired ceramics.

**Figure 7:**
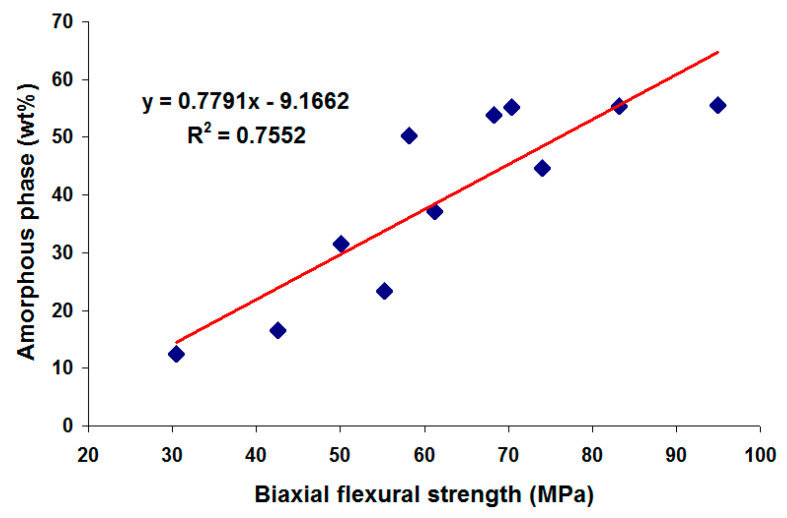
Correlation of the biaxial flexural strength and amorphous phase content of the fired ceramics.

The biaxial flexural strength of the fired ceramics is shown as a function of CFAG content in Figure 6. Initial substitution of kaolin clay by CFAG resulted in significant deterioration in the biaxial flexural strength of the samples, from 94.9 MPa for sample K to 50.1 MPa for sample KC50. This is because the CFAG addition reduced the amount of amorphous phase in the samples after the firing process. The presence of the amorphous phase is crucial to provide better bonding to other components in the samples. An unexpected increase in biaxial flexural strength in sample KC60 (74 MPa) is also observed. This out of trend result can be explained by the increase in amorphous phase in KC60 as the result of quartz vitrification. Figure 7 illustrates the linear relationships between the biaxial flexural strength and amorphous phase content of the samples, which shows a linear correlation of 0.76. The mechanical strength of ceramics is strongly dependent on the microstructural parameters, such as porosity, grain size and shape, crystalline phase composition, relative volume of crystalline and amorphous phases [28]. The fired ceramics prepared in this study are heterogeneous materials, with multiple crystalline phases, different porosities and amorphous content. This explains the relatively low value of the correlation coefficient.

**Figure 8:**
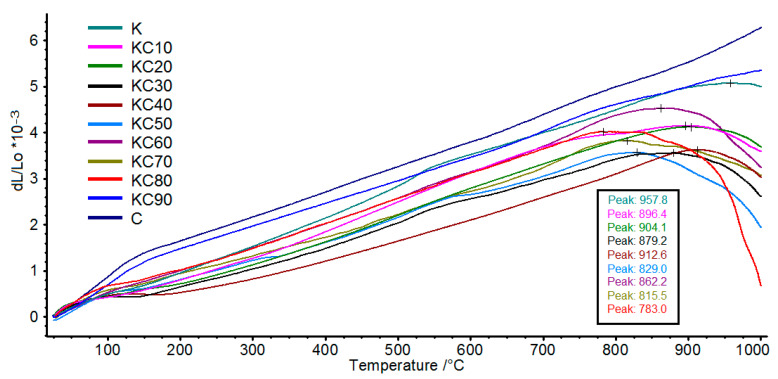
Thermal expansion curves of the fired ceramics at temperatures from 30 °C to 1000 °C. Inset: temperature of maximum expansion of the fired ceramics.

In this study, we have also analyzed the linear thermal expansion of the fired ceramics at temperatures from 30 °C to 1000 °C. The effects of CFAG addition on thermal expansion of the fired ceramics are shown in Figure 8. A small but noticeable thermal expansion change which occurred at around 550 °C can be observed in sample K. Conversely, a greater magnitude of thermal expansion change occurred below 200 °C in sample C. The changes were due to alpha-beta inversion of cristobalite at around 200 °C and alpha-beta inversion of quartz at around 550 °C. This is consistent with the mineralogical composition of the samples shown in Figure 3, which shows that sample K contained a large amount of quartz with no cristobalite, whereas sample C contained a large amount of cristobalite with no quartz. The thermal expansion changes are less pronounced in samples with low content of quartz and cristobalite.

**Figure 9:**
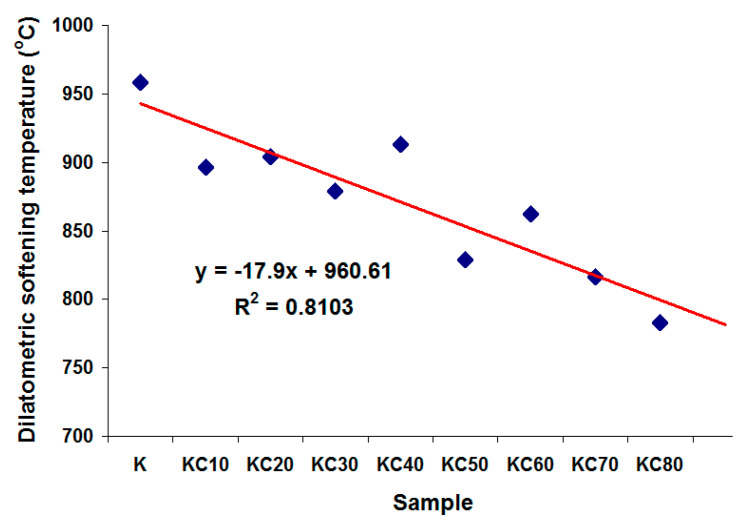
Correlation of the dilatometric softening temperature and CFAG content of the samples.

The refractoriness of ceramics is usually measured by its softening point. The higher the temperature of the softening point, the higher the refractoriness of the ceramic. In this dilatometry analysis, the temperature of maximum thermal expansion, which is referred to as the dilatometric softening temperature, is used to gauge the refractoriness of the fired ceramics. Figure 8 shows that when kaolin clay was gradually replaced by CFAG in the ceramic’s composition, a gradual decrease in the dilatometric softening temperature was observed. Figure 9 illustrates the inverse linear relationships between the dilatometric softening temperature and CFAG content of the samples, which shows a negative linear correlation of 0.81. This can be explained by the high content of alkali metal oxides (K_2_O, CaO) in CFAG. The alkali metal oxides are well-known to create fluxes in ceramic making, which can reduce the working temperature and lower the melting point of ceramics. One should note that samples C and KC90 have surprisingly very high dilatometric softening temperatures (beyond 1000 °C) even though they had higher content of alkali metal oxides. Thus, it can be concluded that the softening effect was dependent on the presence of an adequate amount of kaolin clay.

## 4. Conclusions

The experimental results showed that CFAG has significant impacts on the mineralogy of fired ceramics. The addition of CFAG in the samples increased the mullite and cristobalite content but decreased the quartz and amorphous phase content. Results revealed that when the amount of CFAG addition was below 50 wt %, CFAG behaved as a grog material in the preparation of kaolin clay ceramics. At these amounts, an increase in CFAG addition led to a gradual reduction in the firing shrinkage of the ceramics while at the same time slightly increasing its total porosity. A modest increase in porosity will allow the green body to dry faster and moisture to escape with ease during firing. Lower firing shrinkage could also prevent cracking and distortion. Both of these properties can benefit the preparation of ceramics. The macromorphology study concluded that the addition of CFAG does not affect the dimensional stability of the ceramics at the firing temperature of 1200 °C. However, results also showed that CFAG addition in general has negative effects on the biaxial flexural strength and refractoriness of the ceramics. The low refractoriness of the fired ceramics was due to thermal reaction between alkali metal oxides in CFAG and kaolin clay. Nevertheless, CFA can be considered for grog applications as it is an environmentally sound, economically viable material.

## Figures and Tables

**Table 1:** Chemical compositions of coal fly ash (CFA) and kaolin clay.

Compound	Content (wt %)									
	Al_2_O_3_	SiO_2_	K_2_O	Fe_2_O_3_	TiO_2_	P_2_O_5_	CaO	MgO	Others	LOI
Coal Fly Ash (CFA)	33.51	55.53	3.57	3.36	0.47	0.32	0.31	–	0.13	2.8
Kaolin Clay	34.50	47.50	1.30	0.90	0.30	–	0.03	0.30	0.05	15.12

**Table 2:** Batch composition of the mixtures and the sample names used in the study.

Weight Percentage (wt %)	Sample Name										
	K	KC10	KC20	KC30	KC40	KC50	KC60	KC70	KC80	KC90	C
Kaolin Clay	100	90	80	70	60	50	40	30	20	10	0
CFAG	0	10	20	30	40	50	60	70	80	90	100
